# Prediction of Hematoma Expansion in Hypertensive Intracerebral Hemorrhage by a Radiomics Nomogram

**DOI:** 10.12669/pjms.39.4.7724

**Published:** 2023

**Authors:** Jialin Dai, Dan Liu, Xia Li, Yuyao Liu, Fang Wang, Quan Yang

**Affiliations:** 1 Jialin Dai, Department of Radiology, Yongchuan Hospital of Chongqing Medical University, Chongqing 402160, P.R. China; 2 Dan Liu, Department of Radiology, Yongchuan Hospital of Chongqing Medical University, Chongqing 402160, P.R. China; 3 Xia Li, Department of Radiology, Yongchuan Hospital of Chongqing Medical University, Chongqing 402160, P.R. China; 4 Yuyao Liu, Department of Radiology, Yongchuan Hospital of Chongqing Medical University, Chongqing 402160, P.R. China; 5 Fang Wang Department of Research and Development Shanghai United Imaging Intelligence Co. Shanghai 200232, P.R. China; 6 Quan Yang, Department of Radiology, Yongchuan Hospital of Chongqing Medical University, Chongqing 402160, P.R. China

**Keywords:** Hypertensive intracerebral hemorrhage, Hematoma expansion, Radiomics, Clinical characteristics, Nomogram

## Abstract

**Objective:**

To develop and validate a radiomics-based nomogram model which aimed to predict hematoma expansion (HE) in hypertensive intracerebral hemorrhage (HICH).

**Methods:**

Patients with HICH (n=187) were included from October 2017 to March 2022 in the Yongchuan Affiliated Hospital of Chongqing Medical University. Patients were randomly divided into a training set (n=130) and a validation set (n=57) in a ratio of 7:3. The radiomic features were extracted from the regions of interest (including main hematoma, the surrounding small hematoma(s) and perihematomal edema) in the first CT scan images. The variance threshold, SelectKBest and LASSO (least absolute shrinkage and selection operator), features were selected and the radiomics signature was built. Multivariate logistic regression was used to establish a nomogram based on clinical risk factors and the Rad-score. A receiver operating characteristic (ROC) curve was used to evaluate the generalization of the models’ performance. The calibration curve and the Hosmer-Lemeshow test were used to assess the calibration of the predictive nomogram. And decision curve analysis (DCA) was used to evaluate the prediction model.

**Results:**

Thirteen radiomics features were selected to construct the radiomics signature, which has a robust association with HE. The radiomics model found that blend sign was a predictive factor of HE. The radiomics model ROC in the training set was 0.89 (95%CI 0.82-0.96) and was 0.82 (95%CI 0.60-0.93) in the validation set. The nomogram model was built using the combined prediction model based on radiomics and blend sign, and worked well in both the training set (ROC: 0.90[95%CI 0.83-0.96]) and the validation set (ROC: 0.88[95%CI 0.71-0.93]).

**Conclusion:**

The radiomic signature based on CT of HICH has high accuracy for predicting HE. The combined prediction model of radiomics and blend sign improves the prediction performance.

## INTRODUCTION

Stroke is one of the main causes of death and disability in China, and hypertensive intracerebral hemorrhage (HICH) is the most common form of hemorrhagic stroke.^1^,^2^ According to *Brouwers’s* test, approximately 30% of patients who had an intracerebral hemorrhage (ICH) experienced continued bleeding after the initial event.^3^ Patients with hematoma expansion (HE), a complication of ICH, are at increased risk of poor outcomes (43.5% vs. 5.8%) and reported a higher 90-day Modified Rankin Score (mRS) (67/85, 78.8% vs. 68/172, 39.5%), suggesting increased neurological disability, than patients without HE.^3^,^4^ Early prediction of HE, coupled with rapid implementation of acute interventions, can improve long-term outcomes in this devastating disease.^3^-^5^

A head CT scan is the gold standard for the diagnosis of ICH, and HE is typically diagnosed using the morphology, density, volume and other imaging features of the main hematoma.^6^,^7^ Recent studies have shown that imaging markers such as the island sign, blend sign and surrounding edema of HE have predictive effects beyond the main hematoma.^7^,^8^ In addition, age, gender, blood glucose, blood calcium, the Glasgow Coma Scale (GCS), systolic blood pressure (SBP), and diastolic blood pressure (DBP) provide further prediction of HE.^7^-^10^

To create a prediction model of HE in ICH, we aimed to develop and validate a radiomics-based nomogram, as the nomogram has been widely used as a clinical predictive method in both stroke and ICH.^9^,^10^ The radiomics signature was built by counting the main hematoma and surrounding factors.

## METHODS

We reviewed the clinical data of patients in the EMRS (Electronic Medical Records Systems) and PACS (Picture Archiving and Communication Systems) from October 2017 to March 2022 in the Yongchuan Affiliated Hospital of Chongqing Medical University. A total of 187 patients (63.5 6±11.76 years; range 19-90 years) were identified with HICH by head CT scans in this study.

### Ethical Approval:

The medical research ethics committee of Yongchuan Affiliated Hospital of Chongqing Medical University has approved the study (No. 2022-56, Date: 2022-07-15).

### Inclusion criteria:

- The first head CT scan was performed within 24 hours and re-examined within 24 hours.
- Over 18 years of age, with a history of hypertension or a diagnosis of hypertension during this hospitalization.
- Complete clinical laboratory tests, and no long-term use of anticoagulants.

### Exclusion criteria:

- Patients complicated with other diseases causing secondary cerebral hemorrhage (such as arteriovenous malformations, aneurysm rupture, brain trauma, brain tumor hemorrhage).
- Hemorrhage after infarction.
- Primary intraventricular hemorrhage.
- Poor quality image.

**Supplementary Fig.1:**
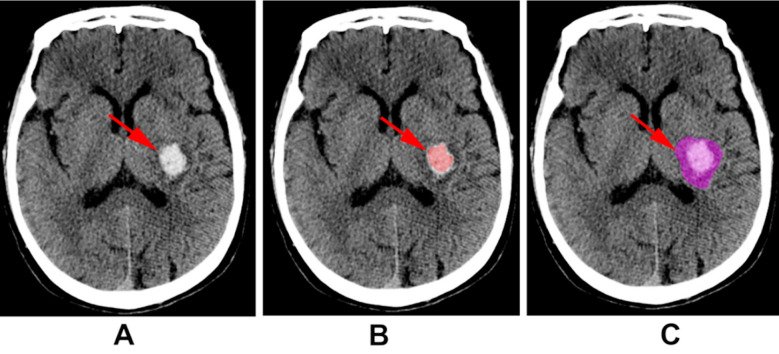
Images of head CT. A: primary image of cerebral hemorrhage. B: region of interest (ROI) of main hematoma and surrounding small hematoma and edema. C: ROI of main hematoma.

HE was defined as a 33%- or 6-ml increase in hematoma volume. The head spiral CT scan was performed using a Philips Brilliance 265 iCT machine. The scanning parameters were set at a tube voltage of 120kV, a tube current of 400mAs, the slice thickness was 5mm, the slice interval was 5mm, the pitch was 0.984-1.375, and the matrix was 512×512. Radiomic feature extraction is described in the supplementary data. All CT images were accessed in DICOM format with a CT image brain window width of 80-100HU and window level of 30-40HU. Imaging data was kept in the uAI research portal (Shanghai United Imaging Intelligence Co., Ltd, version: 21130). The region of interest (ROI) of the main hematoma, surrounding small hematoma and edema of the ICH was determined by two experienced radiologists (Supplementary Fig.1A, B, C). Fifty CT images were randomly selected to determine the inter- and intra-observer agreement of ROI-based feature reproducibility by reader-1 (a radiologist with four years of experience) and reader-2 (a radiologist with 30 years of experience). Reader 1 repeated the same procedure in the one month follow up. An inter- and intra-class correlation coefficient (ICC) greater than 0.75 indicated good agreement of the feature extraction.

A total of 2259 radiomic features were extracted from images which were preprocessed by Z-score normalization to eliminate difference. Methods of dimension reduction, variance threshold, SelectKBest and least absolute shrinkage and selection operator (LASSO), were used to select the best predictive feature. A radiomics signature was calculated for each patient via a linear combination of selected features that were weighted by their respective coefficients. The association of the radiomics signature with HE was first assessed in the training set and then validated in the validation set using a Mann-Whitney U-test.

All processes were run using the uAI research portal (Shanghai United Imaging Intelligence Co., Ltd, version: 21130). A multivariate logistic regression model was performed to identify the independent factors among radiomics signature, clinical variables, and radiographic features to identify HE and NHE in the training set. A radiomics nomogram was constructed based on the multivariate logistic regression model. A radiomics signature was calculated for each patient using the formula constructed in the training set.

### Statistical analysis:

RStudio 3.3.2 and SPSS 26.0 were used for statistical analysis. Measurement data with homogeneity of variance of normal distribution were expressed as mean ± SD, and an independent sample t-test was used to compare differences between groups. Continuous variables with non-normal distribution were expressed as [M (Q1, Q3)], and differences between groups were analyzed by Mann-Whitney U-test. Enumeration data were expressed as numbers and percentages, and differences between groups were compared by chi-square test or Fisher exact test. Binary Logistic Regression was used to establish the prediction model for the indicators with statistical differences. An ROC curve was used to analyze the predictive value of the models. The prediction ability of the nomogram was measured by a calibration curve. The Hosmer Lemeshow (HL) test was used as the model fitting index to judge the gap between the predicted value and the real value. If the p-value is bigger than 0.05, it indicates that there is no significant difference between the predicted value and the real value. The HL test was performed to assess the goodness-of-fit of the nomogram, and a decision curve analysis (DCA) was carried out with the best model. *P* value <0.05 was considered statistically significant.

## RESULTS

**Fig.1:**
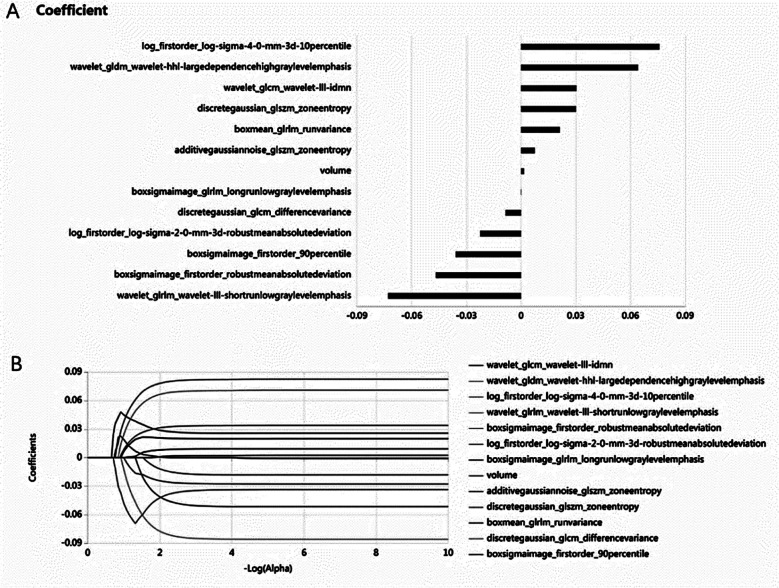
Feature screening process. A: 13 radiomics features selected by LASSO algorithm. B: LASSO path, abscissa is alpha value, ordinate is LASSO coefficient, change trend of each radiomics feature in each solid line.

The baseline clinical and imaging data of the training set and the validation set are shown in Table-I. There was no significant difference in general statistical data between the two groups (*P* > 0.05). A total of 2259 imaging features were extracted from each patient, and 13 robust features were screened out by the variance threshold method, SelectKBest100 and LASSO regression analysis. Radiomics scores of patients were calculated according to the features and their coefficients (Fig.1A). Features with non-zero coefficients were selected and the radiomics score (rad_score) was calculated and converted to a probability (range 0-1) of HE for each patient by using the following formula:


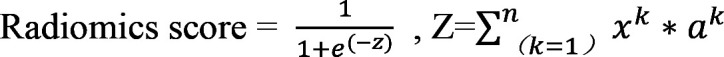


**Table-I:** Comparison of clinical data between training set and validation set.

	Training set (n=130)	Validation set (n=57)	t/χ^2^/z-value	P-value
Age (years)	63.13±11.53	65.09±12.37	1.044	0.298
Gender (male,%)	75 (57.69)	37 (64.91)	0.010	0.992
Time to initial CT (h)	3.00 (2.00,5.00)	3.00 (2.00,4.00)	0.960	0.337
Admission SBP (mmHg)	175.72±27.71	181.25±26.18	1.277	0.203
Admission DBP (mmHg)	102.00±17.72	102.98±17.81	0.348	0.728
Glycated hemoglobin (%)	5.70 (5.40,6.16)	5.74 (5.52,6.20)	1.258	0.262
Intraventricular Hemorrhage (%)	19(14.61)	10(17.54)	0.259	0.611
Island sign (%)	24(18.46)	15(26.32)	1.481	0.224
Blend sign (%)	25(19.23)	16(28.07)	1.809	0.179
GCS	12.00 (10.00,13.00)	12.00 (10.00,13.75)	0.420	0.517
Rad Score	0.34±0.28	0.34±0.25	-0.056	0.956

**Fig.2:**
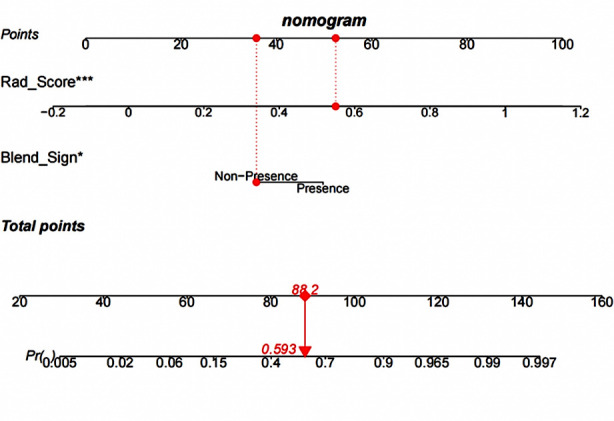
The radiomics nomogram was developed in the training set, with the radiomics signature, and the presence of blend sign.

Where x^k^ represents the selected radiomics features, a^k^ was the respective coefficients, and e refers to the Euler number (e = 2.71828). The 13 features were converted into a radiomics signature displayed in Fig.1B. In the training set, the differences of blend sign and Rad_score were statistically significant (P<0.05) in HE and NHE groups (Tables-II and III). The nomogram model was conducted to visualize the results of the multivariable logistic regression analysis which was generated by the Rad_score and blend sign. As shown in Fig.2, a patients’ imageomics score was 0.03513, and there was no mixed sign. The corresponding score is 88.2 in total, and the probability of the existence of HE is 59.3%.

**Table-II:** Comparison of hematoma expansion and non-hematoma expansion.

	HE(n=45)	NHE(n=85)	t/χ^2^/z-value	P-value
Age (years)	63.60±12.26	62.88±11.19	0.336	0.737
Gender (Male,%)	28 (62.22)	47 (55.29)	0.595	0.440
Time to initial CT (h)	3.00(2.00,4.00)	3.00 (2.00,5.00)	-1.799	0.072
Admission SBP (mmHg)	179.04±32.03	173.95±25.15	0.997	0.321
Admission DBP (mmHg)	104.96±20.81	100.44±15.76	1.388	0.167
Glycated hemoglobin (%)	5.70 (5.43,6.35)	5.71 (5.38,6.10)	0.098	0.922
Intraventricular hemorrhage (%)	19 (42.22)	22 (25.88)	2.806	0.094
Island sign (%)	8 (17.78)	15 (17.65)	0.006	0.940
Blend sign (%)	20 (44.44)	19 (22.35)	5.515	0.019
GCS	9.00 (7.00,10.00)	12.00 (9.00,13.00)	-4.757	<0.001
Rad_score	0.58±0.25	0.21±0.19	-4.766	<0.001

**Table-III:** Risk factors for hematoma expansion in HICHs.

Model 1					Model 2			

	B	OR	95%CI	P	B	OR	95%CI	P
Blend sign	-1.358	0.257	0.073-0.902	0.034	-1.352	0.259	10.075-0.896	0.033
GCS	-1.358	0.849	0.680-1.059	0.147				
Rad score	6.932	1024.520	58.283-18009.435	<0.001	7.644	2088.791	131.823-33097.831	<0.001

**Fig.3:**
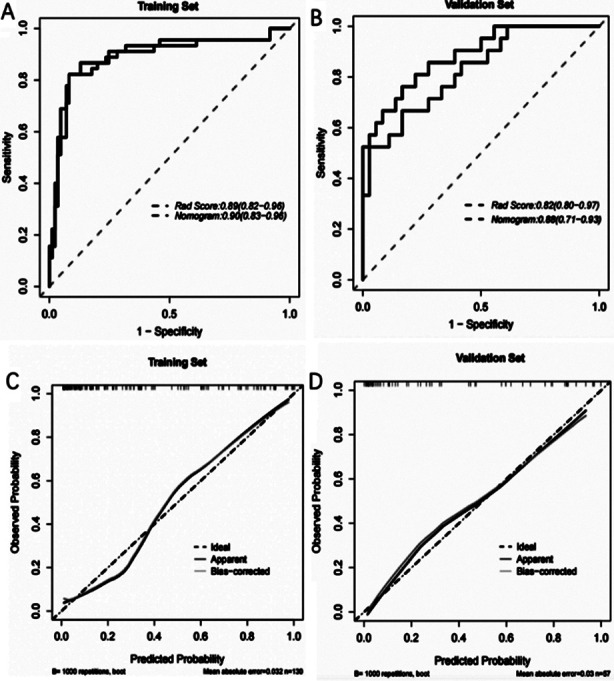
A) Pairwise comparison of receiver operating characteristic (ROC) curves for Radscore and the nomogram model in the validation set. B) Pairwise comparison of ROC curves for Radscore and the nomogram model in the training set. C) Calibration curve of the nomogram model in the training set. D) Calibration curve of the nomogram model in the validation set.

The calibration curve of the radiomics nomogram for the probability of HE in ICH demonstrated good agreement between prediction and observation in the two sets (Fig.3A and B). The Hosmer-Lemeshow test (Fig.3C and D) yielded a non-significant statistic in both the training and validation set, which suggested that there was no departure from a perfect fit. The C-index for the prediction nomogram was 0.90(95% CI 0.83-0.96) in the training set and 0.88(95%CI 0.71-0.93) in the validation set.

### Clinical Use:

**Fig.4:**
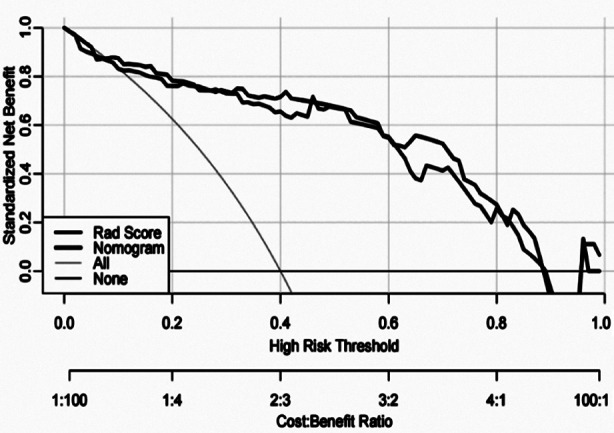
DCA for the radiomics-based nomogram. The y-axis represented the net benefit.

The decision curve analysis (DCA) showed that a threshold probability of a patient or doctor of 10%, using the radiomics nomogram to predict HE, adds more benefit than either the treat-all-patients scheme or the treat-none scheme (Fig.4). Within this range, net benefit was comparable, with several overlaps, based on the radiomics nomogram and the model with histologic grade integrated.

## DISCUSSION

In this study, the occurrence of HE in patients with HICH was predicted by our combined radiomic nomogram model. This nomogram was built with 13 imageomic features screened by the Lasso Logistic regression model, and the corresponding weighting coefficient. In addition to one shape feature, 12 of the 13 selected features in the imageomics model are microscopic information that cannot be obtained by the naked eye. The shape feature describes the geometric characteristics of RIO. The shape feature in this study was the volume of voxels. The existence of voxel volume further verifies that HE is closely related to the volume of hematoma and edema in patients with HICH.^10^,^11^ In addition, the 10th Percentile, 90th Percentile, GLDM, GLCM, GLSZM and other histone features are also included.^12^ These features, due to the differences in cell structure, are shown as the spatial relationship and density differences between CT image pixels.^12^,^13^ Imagomics converts the observable and unobservable image information into deep level features for quantitative analysis to achieve repeatability and stability.^11^,^13^ The AUC in the training group was 0.89 (0.82-0.96), and that in the validation group was 0.82 (0.80-0.97). Similar to the prediction efficiency of only drawing the hematoma ROI, our method can also accurately predict the occurrence of HE, solving the problem of assessing an irregular hematoma, which is difficult to draw.^14^

In this study, blend sign was screened as a clinical, independent factor, which was first proposed by Li Q et al.^6^ and was based on the observation of non-enhanced CT scanning. The presence of blend sign and CTA spot sign were independent predictors of hematoma growth.^15^,^16^ Although CTA spot sign and leakage sign are of high value in predicting the occurrence of E in patients with primary cerebral hemorrhage, they have shortcomings such as excessive radiation dose, high cost, and risk of allergic reaction.^13^,^17^ Therefore, they cannot be used as the first choice for examination, while the blend sign is convenient and suitable for general use.^9^,^11^,^12^ Hematoma density is affected by its components, specifically, hemoglobin is an important factor that determines the hematoma density on a CT scan.^11^,^13^,^18^ When blood clots, the hematoma appears as high density on a CT scan. When there is active bleeding, the hematoma tends to be a lower density than the clot. The mixed blood at different bleeding times leads to the appearance of mixed syndrome. Sporns PB et al.^19^ found that both blend sign and spot sign can predict early HE, but also early neurological deterioration, suggesting the value of blend sign may be higher than that of CTA spot sign.

By adding independent clinical factors based on the radiomic model, we can easily find that the combined radiomic model shows good predictive effectiveness. The ROC of the training group was 0.90 (95CI% 0.83-0.96), and the ROC of the validation group was 0.88 (95CI% 0.71-0.93). Our combined prediction model of radiomics and blend sign had higher predictive performance than the previous model, constructed by only using radiomics. Therefore, the combined model may be a better choice for predicting HE in patients with HICH.^10^,^14^,^19^,^20^

### Limitations:

This retrospective analysis used data from a single institution, with a small sample size. Furthermore, the sample sizes of the HE and NHE groups are unbalanced, and as such there may be selection bias.

## CONCLUSION

The combined prediction model of radiomics and blend sign successfully predicted HE in patients with HICH. The results presented here suggest that this assessment model can be used to guide early clinical diagnosis to reduce the mortality and poor prognosis of patients with HICH. Specifically, this assessment model can aid in the prediction of whether patients with HICH will experience HE.

### Authors’ contributions:

**JD:** Conceived and designed the study.

**DL, XL, YL, FW** and **QY:** Collected the data and performed the analysis.

**JD:** Was involved in the writing of the manuscript, is responsible for the integrity of the study.

All authors have read and approved the final manuscript.

The authors declare that the research was conducted in the absence of any commercial or financial relationships that could be construed as a potential conflict of interest.
